# Time-varying reproductive number estimation for practical application in structured populations

**DOI:** 10.1515/em-2024-0020

**Published:** 2025-01-06

**Authors:** Erin Clancey, Eric T. Lofgren

**Affiliations:** Paul G. Allen School for Global Health, Washington State University, Pullman, WA, USA

**Keywords:** time-varying reproductive number, population structure, EpiEstim, generation interval distribution, simulation

## Abstract

**Objectives:**

EpiEstim is a popular statistical framework designed to produce real-time estimates of the time-varying reproductive number, ${ℛ}_{t}$. However, the methods in EpiEstim have not been tested in small, non-randomly mixing populations to determine if the resulting ${\overset{ˆ}{ℛ}}_{t}$ estimates are temporally biased. Thus, we evaluate the temporal performance of EpiEstim ${\overset{ˆ}{ℛ}}_{t}$ estimates when population structure is present, and then demonstrate how to recover temporal accuracy using an approximation with EpiEstim.

**Methods:**

Following a real-world example of a COVID-19 outbreak in a small university town, we generate simulated case report data from a two-population mechanistic model with an explicit generation interval distribution and expression to compute true ${ℛ}_{t}$. To quantify the temporal bias, we compare the time points when true ${ℛ}_{t}$ and estimated ${\overset{ˆ}{ℛ}}_{t}$ from EpiEstim fall below the critical threshold of 1.

**Results:**

When population structure is present but not accounted for ${\overset{ˆ}{ℛ}}_{t}$ estimates from EpiEstim prematurely fall below 1. When incidence data is aggregated over weeks the estimates from EpiEstim fall below the critical threshold at a later time point than estimates from daily data, however, population structure does not further affect timing differences between aggregated and daily data. Last, we show it is possible to recover the correct timing when by using the lagging subpopulation outbreak to estimate ${\overset{ˆ}{ℛ}}_{t}$ for the total population with EpiEstim.

**Conclusions:**

${ℛ}_{t}$ is a key parameter used for epidemic response. Since population structure can bias ${ℛ}_{t}$ near the critical threshold of 1, EpiEstim should be prudently applied to incidence data from structured populations.

## Introduction

The time-varying reproductive number, ${ℛ}_{t}$, is the average number of secondary infections generated by a single individual over the infectious period at time t [1,2]. ${ℛ}_{t}$ is a key epidemic parameter that quantifies the transmissibility of a pathogen over the course of an epidemic [2]. Monitoring the reproductive number as an outbreak progresses can provide instantaneous feedback to public health officials on the effectiveness of control measures [1]. Specifically, the reproductive number indicates whether an epidemic is growing (${ℛ}_{t}>1$), falling (${ℛ}_{t}<1$), or remaining at equilibrium (${ℛ}_{t}=1$). Therefore, predicting when ${ℛ}_{t}$ falls below the critical threshold of 1 in real-time has important implications for policy decisions, especially as it was commonly used during the COVID-19 pandemic as a signal for easing existing interventions [1, 3, 4].

Many methods to estimate ${ℛ}_{t}$ in real-time are available [e.g., 1, 5–9]. Among the most popular is the EpiEstim framework developed by Cori et al. [1] and freely available as an R package [10, 11]. EpiEstim is favored because it is simple to implement, requiring only incidence time series and the generation interval distribution (often approximated with the serial interval distribution), and gives reliable near real-time estimates of ${ℛ}_{t}$ in many contexts [12]. In addition to daily incidence data, EpiEstim can utilize temporally aggregated data even when the time window of incidence reporting is longer than the mean generation interval (e.g., when incidence is reported over weekly intervals or aggregated to reduce administrative noise, such as the effects of the weekends) [3]. Because EpiEstim relies on a simple model, the method makes several strict assumptions. For example, EpiEstim assumes infectiousness begins at symptom onset, testing rate is constant over the course of an epidemic, transmission rate is constant over smoothing windows, and importation of cases does not occur [1]. Despite these limitations, EpiEstim has been well tested in large, randomly mixing populations and produces reliable estimates of ${ℛ}_{t}$ for both daily incidence data [1, 12] and temporally aggregated data [3]. However, these methods have not been evaluated for application to smaller populations that are highly structured with non-random mixing occurring between subpopulations.

Population structure has long been recognized to play an important role in the spread of infectious disease [13, 14]. Clearly, not all people mix homogeneously within larger populations. Yet our empirical and theoretical methods often ignore heterogeneous mixing because increasing model realism requires increasing data requirements and model complexity [13–18]. Nonetheless, when individual variation in infectiousness is not accounted for, population level estimates such as ${ℛ}_{0}$ can be unreliable [14, 19, 20]. Specific to EpiEstim, non-random mixing and/or superspreading events can bias the temporal accuracy of ${ℛ}_{t}$ estimation. For example, Cori et al. [1] document ${ℛ}_{t}$ estimates falling below 1 before successive incidence peaks occurred in pandemic influenza in Baltimore, Maryland in 1918 and while incidence was still high in the SARS outbreak in Hong Kong in 2003. Temporal inaccuracy of ${ℛ}_{t}$ estimation, particularly when the date that ${ℛ}_{t}$ falls below 1 is misspecified, has consequences for understanding how behavioral changes affect transmission or policy decisions to cease control measures [12].

Here, we evaluate the temporal performance of ${ℛ}_{t}$ estimation in the EpiEstim framework when population structure is present in small populations. We use simulated data from a two-population mechanistic model and estimate the time point at which the estimate of ${ℛ}_{t}$ falls below 1 assuming the structure is “hidden” from the user of EpiEstim. Then, we show how to recover accurate timing of ${ℛ}_{t}$ estimates from EpiEstim if data on incidence by subpopulation is available. To connect our assessment of EpiEstim to real data, we build synthetic epidemics mimicking the fall 2020 COVID-19 outbreak in Whitman County, Washington, USA. Whitman County harbors two distinct populations, the Whitman County community residents centered around the city of Pullman, WA, and the Washington State University (WSU) resident student body. Like other COVID-19 outbreaks in university towns (e.g., [21]), evidence suggests non-random mixing resulted in sequential COVID-19 outbreaks in Whitman County during fall 2020 [22] where the incidence in each subpopulation is clearly delineated. Our specific analyses include assessing the temporal accuracy of EpiEstim when ${ℛ}_{t}<1$ for daily incidence data and weekly aggregated data. Since we use simulated data and EpiEstim requires the generation or serial interval distribution as input, we also offer a framework to simulate a two-population compartment model and derive the generation interval distribution from this model. Last, we show that if incidence is known within population groups (e.g., community member vs. university student), EpiEstim estimates from the subpopulation with the lagging outbreak can be used as a non-biased approximation to the timing of ${ℛ}_{t}$ falling below 1.

## Methods

### Real data with population structure

We motivate our two-population model and synthetic epidemics with daily COVID-19 incidence reported to Whitman County, Washington, USA during fall 2020. Located in an agricultural area of southeastern Washington, Whitman County is home to Washington State University (WSU), a public research land-grant university, and the city of Pullman. Although the Whitman County community residents and WSU student populations overlap geographically, population mixing does not occur randomly. Students are largely concentrated in housing on or near the WSU campus, and community members live in the city of Pullman or are dispersed throughout Whitman County. During the fall semester all courses were fully remote, however, many students returned to the Pullman campus. COVID testing was available for both community residents and WSU students, and lockdown measures and masking requirements were already in place at both WSU and Pullman city public spaces and many private businesses at the beginning of case reporting. Thus, transmission rates were very likely near constant throughout the outbreak. Even with interventions in place, Whitman County reported an outbreak of COVID-19 within the student and subsequently the community populations with one of the highest rates reported in Washington State at the time [22]. The epidemic time series data we present here begins on August 17, 2020, the week prior to the beginning of the semester when most students returned to campus, and reports until December 27, 2020 (days 230–362 on the Julian calendar). We implement EpiEstim on the daily case report data, weekly aggregated data, and separately for the WSU student and Whitman County community subpopulations.

### Two-population model

To study the effects of population structure on ${ℛ}_{t}$ estimates from EpiEstim, we use a simple metapopulation model that has explicit intra- and inter-community interactions [16] similar to the structure present in Whitman County. Specifically, we use a two-population Susceptible-Exposed-Infected-Recovered (SEIR) ordinary differential equations (ODE) model framework:

(1a) $${\dot{S}}_{i}=-S_{i}\left(\beta_{i}I_{i}+\beta_{ij}I_{j}\right)$$

(1b) $${\dot{S}}_{j}=-S_{j}\left(\beta_{j}I_{j}+\beta_{ij}I_{i}\right)$$

(1c) $${\dot{E}}_{i}=S_{i}\left(\beta_{i}I_{i}+\beta_{ij}I_{j}\right)-\sigma E_{i}$$

(1d) $${\dot{E}}_{j}=S_{j}\left(\beta_{j}I_{j}+\beta_{ij}I_{i}\right)-\sigma E_{j}$$

(1e) $${\dot{I}}_{i}=\sigma E_{i}-\gamma I_{i}$$

(1f) $${\dot{I}}_{j}=\sigma E_{j}-\gamma I_{j}$$

(1g) $${\dot{R}}_{i}=\gamma I_{i}$$

(1h) $${\dot{R}}_{j}=\gamma I_{j}.$$

We assume the latency rate (σ) and recover rate (γ) are constant and equivalent in both populations. All transmission parameters are constant throughout the epidemic, where cross-transmission from i→j is equal to i←j such that $\beta_{ij}=\beta_{ji}$, and $\beta_{j}\geq \beta_{i}\geq \beta_{ij}$. Population sizes remain constant throughout the duration of the epidemic, with no substantial loss to death or migration. However, we also assume that subpopulation sizes are unequal, but each subpopulation size is large enough to make a significant contribution to the total population size. We chose values for the relative subpopulation sizes based on the sizes of the WSU student and Whitman County Community subpopulations. Definitions of parameters and their symbols are given in Table 1.

#### Specifying true ${ℛ}_{t}$

We derive the time-varying reproductive number directly from our ODE model (Equations (1a)–(1h)) by finding ${ℛ}_{0}$ using the next generation method [23]. When the population is at its disease free equilibrium (DFE), we assume the number of susceptible individuals in each population is equal to each subpopulation size such that $S_{0_{i}}=N_{i}$ and $S_{0_{j}}=N_{j}$. Then we specify ${ℛ}_{t}$ by allowing the number of susceptibles in each subpopulation to vary over time $\left\{S_{i}(t),S_{j}(t)\right\}$ starting at time t=0 to get

(2) $${ℛ}_{t}=\frac{S_{i}(t)\beta_{i}+S_{j}(t)\beta_{j}+\sqrt{{\left(S_{i}(t)\beta_{i}-S_{j}(t)\beta_{j}\right)}^{2}+4S_{i}(t)S_{j}(t)\beta_{ij}^{2}}}{2\gamma }.$$

Equation (2) gives us an exact calculation for ${ℛ}_{t}$ that can be used to compare to the time-varying reproductive number estimates from EpiEstim, which we denote ${\overset{ˆ}{ℛ}}_{t}$.

Time varying reproductive numbers $\left\{{ℛ}_{i_{t}},{ℛ}_{j_{t}}\right\}$ can also be calculated for each subpopulation using the expression ${ℛ}_{t}=\beta S(t)D$, where D is the duration of infection [12]. Given the inter-community mixing matrix from our model in Equations (1a)–(1h) is

(3) $$M=\left[\begin{array}{ll} \beta_{i} & \beta_{ij}\\ \beta_{ij} & \beta_{j} \end{array}\right],$$

the vector ${\left\{S_{i}(t),S_{j}(t)\right\}}^{\prime }$ and constant during of infection $\gamma^{-1}$, the exact subpopulation time varying reproductive numbers can written as a system of equations that rely on the depletion of susceptible individuals across populations:

(4) $$\left[\begin{array}{l} {ℛ}_{i_{t}}\\ {ℛ}_{j_{t}} \end{array}\right]=\left[\begin{array}{l} \beta_{i}S_{i}(t)+S_{j}(t)\beta_{ij}\\ \beta_{j}S_{j}(t)+S_{i}(t)\beta_{ij} \end{array}\right]\gamma^{-1}.$$

We also compare the conditional subpopulation time varying reproductive numbers from Equation (4) to the subpopulation estimates from EpiEstim, likewise denoted ${\overset{ˆ}{ℛ}}_{i_{t}}$ and ${\overset{ˆ}{ℛ}}_{j_{t}}$.

#### Specifying the generation interval distribution

To estimate ${\overset{ˆ}{ℛ}}_{t}$, a user of EpiEstim must supply the mean and standard deviation of the generation interval, or more often in practice the mean and standard deviation of the serial interval distribution is used as an approximation [1, 3, 12]. To test EpiEstim in structured populations without introducing bias by approximating with the serial interval distribution [24], we derive the generation interval distribution directly from our compartment model (Equations (1a)–(1h)) following the mathematical framework presented in Champerdon et al. [25].

Champerdon et al. [25] present a unique result in mathematical epidemiology by explicitly deriving the link between the intrinsic generation interval distribution, g, used in renewal equation models and SEIR compartmental models with Erlang distributed latent and infectious periods. We follow their logic for our two-population compartment model with only one latent stage and one infectious stage that occur simultaneously in subpopulations {i,j}. We let F(τ) be the total probability of drawing a random individual from either infected compartment $\left\{I_{i},I_{j}\right\}\tau$ units of time after being infected. Using the expressions for $F_{k}$ from the system of ODEs in Appendix A.2 from Champredon et al. [25], we formulate an expression for F (i.e. $F_{k=1}$) specific to our two-population model:

(5) $$F(\tau )=\pi_{i}\frac{\sigma_{i}\left(e^{-\sigma_{i}\tau }-e^{-\gamma_{i}\tau }\right)}{\sigma_{i}-\gamma_{i}}+\pi_{j}\frac{\sigma_{j}\left(e^{-\sigma_{j}\tau }-e^{-\gamma_{j}\tau }\right)}{\sigma_{j}-\gamma_{j}},$$

where $\left\{\pi_{i},\pi_{j}\right\}$ are the proportions each subpopulation contributes to the total population size. Since we explicitly assume $\sigma_{i}=\sigma_{j}=\sigma$ and $\gamma_{i}=\gamma_{j}=\gamma$, Equation (6) reduces to

(6) $$F\left(\tau \right)=\frac{\sigma \left(e^{-\sigma \tau }-e^{-\gamma \tau }\right)}{\sigma -\gamma },$$

as given in Champredon et al. [25] when k=1. Further, the intrinsic infectiousness of individuals who have been infected for length of time τ is βF(τ), and we obtain the intrinsic generation interval distribution following [25] by normalizing with ${ℛ}_{0}$ to get an expression for g,

(7) $$g(\tau )=\frac{\beta F(\tau )}{{ℛ}_{0}}.$$

In our model, β in Equation (7) is the composite transmission rate within and across subpopulations. We obtain our composite β by finding the dominant eigenvalue of the inter-community mixing matrix [see [13] in Equation (3) to get

(8) $$\beta =\frac{\beta_{i}+\beta_{j}+\sqrt{{\left(\beta_{i}-\beta_{j}\right)}^{2}+4\beta_{ij}^{2}}}{2}.$$

Now we can use Equation (7) to find the intrinstic generation interval distribution. We use ${ℛ}_{0}$ given by Equation (2) when the DFE is defined by the proportion of susceptible individuals in each population instead of the total. Specifically, $\frac{S_{0_{i}}}{N_{i}}=1$ and $\frac{S_{0_{j}}}{N_{j}}=1$. Finally, the intrinsic generation interval dsitribution for our two-population model in Equations (1a)–(1h) is

(9) $$g(\tau )=\frac{\sigma \gamma }{\sigma -\gamma }\left(e^{-\gamma \tau }-e^{-\sigma \tau }\right),$$

with mean

(10) $$𝔼[\tau ]=\frac{1}{\sigma }+\frac{1}{\gamma },$$

and variance

(11) $$𝕍[\tau ]=\frac{1}{\sigma^{2}}+\frac{1}{\gamma^{2}}.$$

This result for a two-population SEIR compartment model is the same expression given in Table 1 in Champredon et al. [25] when the latency and recovery rates are the same for the subpopulations. All mathematical analyses were performed in Wolfram Mathematica 13.1 [26].

### Synthetic data with population structure

To quantify the effect of hidden population structure on the timeliness of ${ℛ}_{t}$ estimation using EpiEstim, we use data simulated from our two-population model (Equations (1a)–(1h)). We generate synthetic epidemics similar to the COVID-19 outbreak that occurred in Whitman County during fall 2020 (Table 1). We simulate our model (Equations (1a)–(1h)) using Euler’s stochastic method within the pomp R package framework [11, 27] using a time step t of one day. Daily incidence is recorded in each population by tallying individuals that move from an exposed $\left\{E_{i},E_{j}\right\}$ to an infected $\left\{I_{i},I_{j}\right\}$ compartment (at symptom onset) at the end of each day. Cases are reported daily for each subpopulation by sampling from total daily incidence. To sample from daily incidence we use an alternative parameterization of the negative binomial that relies on the mean and dispersion parameter:

(12) $$Reports(t)∼NegBin\left(\rho \;C_{i}(t),k\right)+NegBin\left(\rho \;C_{j}(t),k\right).$$

Here, ρ is the testing rate and $\left\{C_{i},C_{j}\right\}$ are true daily incidence in each subpopulation at time t, which comprise the mean, and k is the dispersion parameter. We use the negative binomial distribution, with its separate variance term, to model case reports from small populations. We assume that testing rate is constant and that cases are reported at symptom onset, to give a realistic delay in reporting from disease onset. Table 1 gives the symbols, definitions, and values (or range of values) used in all simulations. True incidence and case reports are given for the total population as the sum of the incidence and sum of the case reports from the two subpopulations, respectively. We generate 100 synthetic epidemics for each parameter combination in Table 1 to demonstrate the bias in temporal accuracy of the estimates from EpiEstim when population structure is not considered. All simulations were performed in R [11].

### ${ℛ}_{t}$ estimation with EpiEstim

The general method implemented in the EpiEstim R package relies on the renewal equation to estimate

(13) $${\overset{ˆ}{ℛ}}_{t}=\frac{i(t)}{\sum_{\tau =1}^{t}i(t-\tau )g(\tau )},$$

where i(t) is the total number of infection incidents at time t in days [1, 3, 12]. EpiEstim estimates the instantaneous reproduction number which corresponds to the time-varying reproductive number and both are denoted as ${ℛ}_{t}$ [1]. Thus, to obtain estimates for ${\overset{ˆ}{ℛ}}_{t}$ using the EpiEstim package [10], we must supply a time series dataset of incidence, values for the mean and standard deviation for the generation interval distribution, and specify the size of the smoothing window (t−τ). We use case report data reported to Whitman County or generated from our synthetic COVID-19 epidemics, and Equations (10) and (11) to calculate the mean and standard deviation of the generation interval distribution with the values in Table 1. Within the smoothing window, EpiEstim assumes the instantaneous reproductive number remains constant and quantifies the average transmissibility per time window [1]. EpiEstim recommends using weekly time windows as the default because ${\overset{ˆ}{ℛ}}_{t}$ can be highly variable when the size of the smoothing window is small and often case reports have weekend effects [3]. Also as the default, EpiEstim reports ${\overset{ˆ}{ℛ}}_{t}$ on the last time step of the window to align with last day of the incidence time series [12]. Although increasing the smoothing window leads to more precise estimates [1], larger smoothing windows reported on the last day of the window lead to ${\overset{ˆ}{ℛ}}_{t}$ estimates that lag true values [12]. Since our daily case reports are random draws from a negative binomial, we necessitate the use of a smoothing window. We use the EpiEstim recommended default weekly smoothing window since this time window is likely to be most commonly applied. Then, for more accurate time reporting when there is a known latency period and because transmissibility is assumed to be constant over the smoothing window, we report ${\overset{ˆ}{ℛ}}_{t}$ at the beginning of the time window.

Another caveat with the method implemented by EpiEstim (Equation (13)) is the time window of incidence reporting must be in the same time units as the generation or serial interval distribution [3]. Nash et al. [3] extend EpiEstim’s applicability to coarsely aggregated data by implementing an expectation-maximisation algorithm to reconstruct daily incidence from temporally aggregated data and then estimate ${\overset{ˆ}{ℛ}}_{t}$. To test the methods of Nash et al. [3] in structured populations we provide EpiEstim with a weekly time series dataset by aggregating our simulated daily case reports over weekly windows.

Last, we estimate the time-varying reproductive numbers $\left\{{\overset{ˆ}{ℛ}}_{i_{t}},{\overset{ˆ}{ℛ}}_{j_{t}}\right\}$ for each subpopulation with EpiEstim where $\left\{{Reports}_{i}(t),{Reports}_{j}(t)\right\}$ are the number of infection incidents reported within each subpopulation at time t in days. Estimating ${\overset{ˆ}{ℛ}}_{t}$ for subgroups requires incidence is reported by group affiliation. The methods implemented in EpiEstim also require a minimum of 12 cases be recorded before estimation should proceed [1]. Therefore, proper estimation from the subpopulation with the lagging outbreak must begin at a later date than in the subpopulation with the leading outbreak. Using the same simulated data, we compare the timing when ${\overset{ˆ}{ℛ}}_{t}<1$ of the subpopulation with the lagging outbreak to the timing from true ${ℛ}_{t}$ for the total population.

## Results

**Figure 1:**
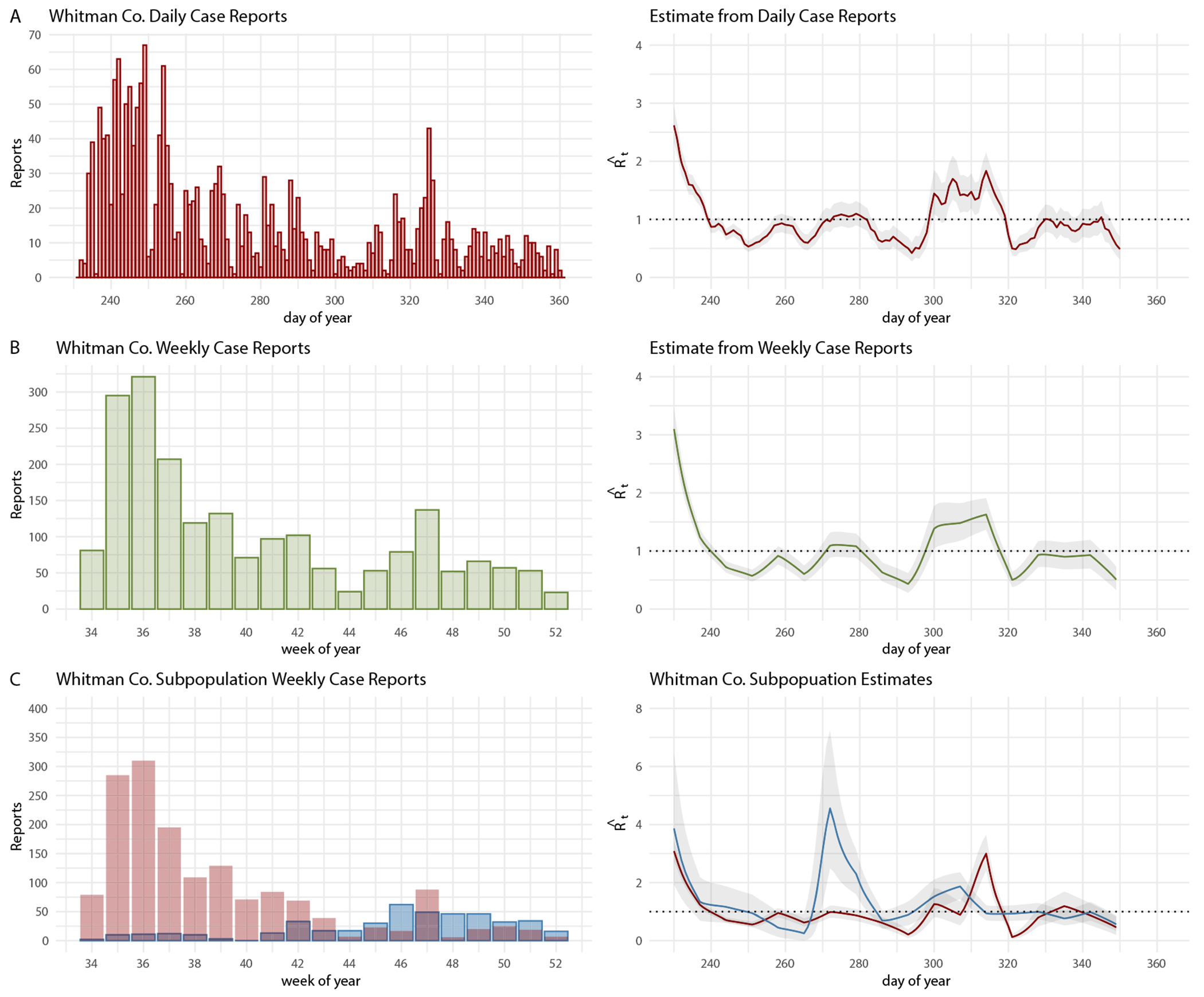
Whitman Co. fall 2020 COVID-19 case reports and ${\overset{ˆ}{ℛ}}_{t}$ with 95 % credible intervals computed from EpiEstim from (A) daily reports, (B) reports aggregated by weeks, and (C) weekly reports separated into WSU student (red) and Whitman Co. community (blue) subpopulations.

We begin our analyses with a demonstration of time-varying reproductive number estimation with EpiEstim using COVID-19 incidence reported to Whitman County, Washington, USA. Figure 1 shows daily and weekly COVID-19 case reports and corresponding ${\overset{ˆ}{ℛ}}_{t}$ estimates from EpiEstim. ${\overset{ˆ}{ℛ}}_{t}$ crosses below the threshold $\left({\overset{ˆ}{ℛ}}_{t}<1\right)$ on day 240 for daily case reports, weekly case reports, and for the WSU student subpopulation (Figure 1). The Whitman County community case reports highlight an issue that arises interpreting ${\overset{ˆ}{ℛ}}_{t}$ estimates from EpiEstim when observed case counts are low. In the community subpopulation the observed case counts during drop to zero counts in week 40 and then return to 13 cases reported in week 41. Estimates from EpiEstim are unreliable when cases drop below 11 reports [1], and therefore the spike in ${\overset{ˆ}{ℛ}}_{t}$ in the community beginning during week 41 (on day 279) cannot be distinguished from stochastic fluctuations in low number of case counts. Beginning in week 41, however, weekly case numbers are greater than 11 and therefore we begin interpreting ${\overset{ˆ}{ℛ}}_{t}$ after week 41. As such, we conclude ${\overset{ˆ}{ℛ}}_{t}$ crosses below the threshold (${\overset{ˆ}{ℛ}}_{t}<1$) on day 285 in the Whitman County community subpopulation (Figure 1C). For this incidence dataset, the Whitman County community subpopulation lags behind the outbreak in the WSU student subpopulation by 45 days.

**Figure 2:**
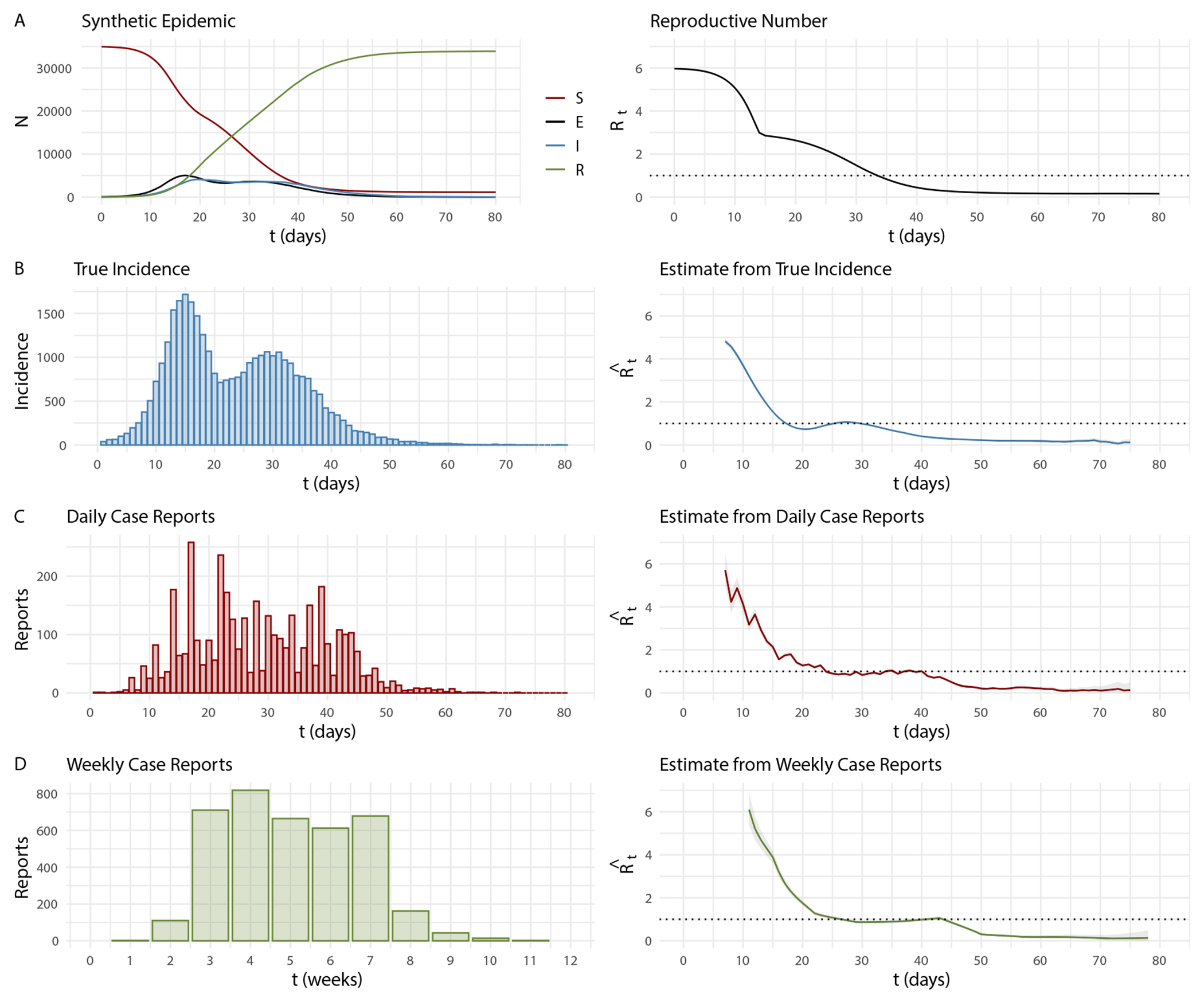
Example of a synthetic COVID-19 outbreak in a structured population with parameter values given in Table 1 where $\beta_{j}=12\times 10^{-5}$ and $\beta_{ij}=1\times 10^{-8}$. (A) Synthetic epidemic and true ${ℛ}_{t}$ curves. (B) True incidence and corresponding ${\overset{ˆ}{ℛ}}_{t}$ with 95 % credible interval computed from EpiEstim. (C) Daily case reports sampled from true incidence and corresponding ${\overset{ˆ}{ℛ}}_{t}$ computed from EpiEstim. (D) Weekly case reports aggregated from daily reports and corresponding ${\overset{ˆ}{ℛ}}_{t}$ computed from EpiEstim.

Because we are interested in quantifying the timing bias from EpiEstim, we present results from simulated data where we know the true time-varying reproductive number, ${ℛ}_{t}$. Figure 2 is an example of one synthetic epidemic from which we generate true incidence, daily and weekly case reports, and estimate ${\overset{ˆ}{ℛ}}_{t}$ using EpiEstim assuming population structure is “hidden”. From the synthetic epidemic in Figure 2, the true ${ℛ}_{t}<1$ occurs on day 34, ${\overset{ˆ}{ℛ}}_{t}<1$ from true incidence, daily case reports, and weekly aggregated case reports occur on days 18, 24, and 27, respectively.

**Figure 3:**
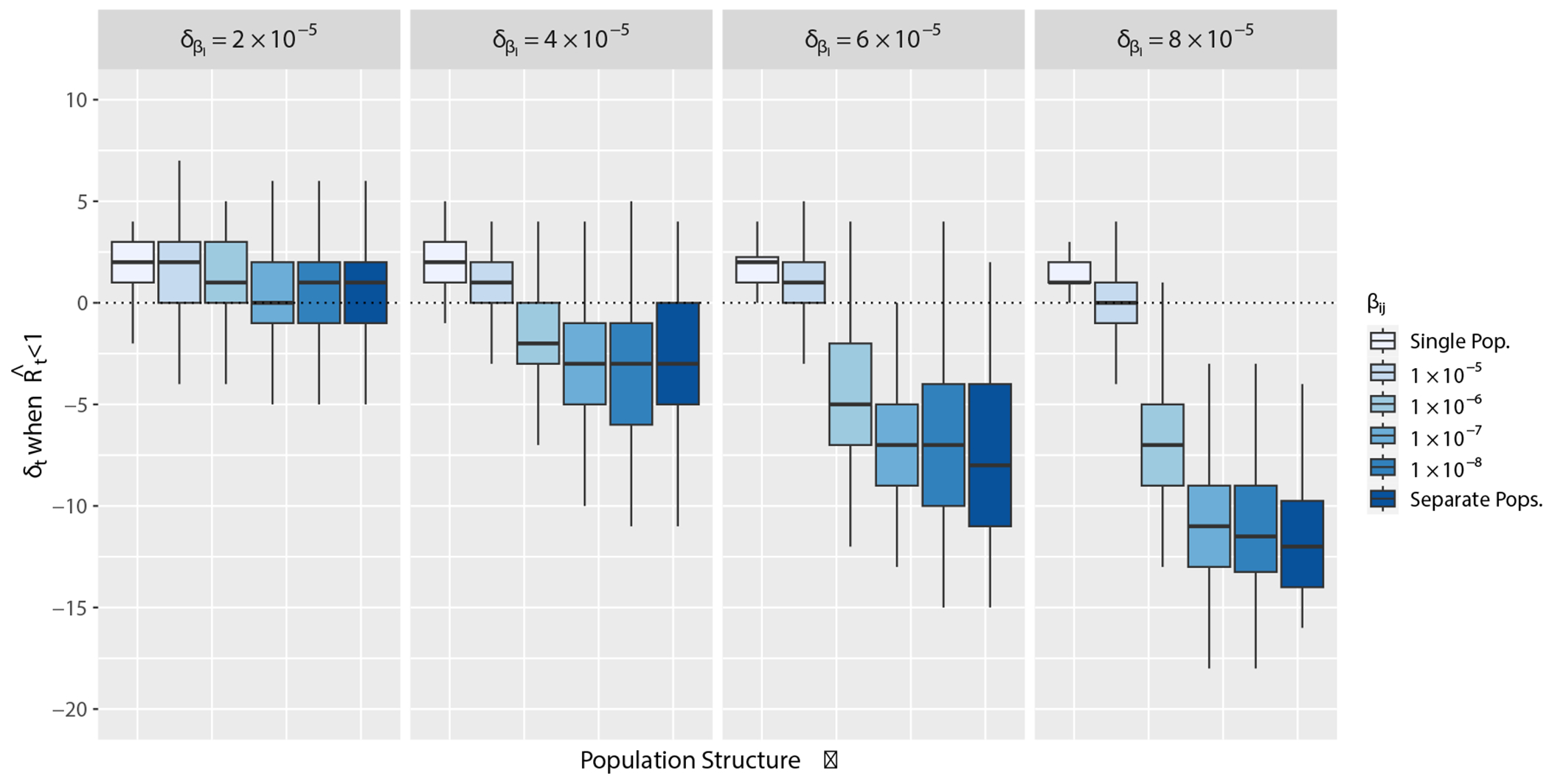
Results from simulated data computing true ${ℛ}_{t}$ and ${\overset{ˆ}{ℛ}}_{t}$ with EpiEstim over varying degrees of population structure. Population structure varies along the x-axis by increasing $\delta_{\beta_{i}}$, the difference $\beta_{j}-\beta_{i}$, and $\beta_{ij}$, the rate of mixing. Within each panel, the lightest colored box represents a single, randomly mixing population where $\delta_{\beta_{i}}=0$ and $\beta_{ij}=\beta_{i}=\beta_{j}$, and the colored boxes correspond to different values of $\beta_{ij}$ or $\beta_{ij}=0$ for separate populations. $\delta_{t}$ on the y-axis is the difference in time points (t) in days of the epidemic time series between ${\overset{ˆ}{ℛ}}_{t}<1$ minus ${ℛ}_{t}<1$.

To evaluate the performance of EpiEstim when applied in small, structured populations, we first quantify the bias in the timeliness of ${\overset{ˆ}{ℛ}}_{t}$ estimated from daily case report data compared to true ${ℛ}_{t}$ computed from Equation (2) from our synthetic epidemics. We measure bias by calculating $\delta_{t}$, which is the difference in time points (t) in days between ${\overset{ˆ}{ℛ}}_{t}<1$ minus ${ℛ}_{t}<1$. The baseline bias between measurements from our model and from EpiEstim in a single, randomly mixing population has a median difference of $\delta_{t}=2$ days. This baseline bias is present because case reporting at the time of symptom onset instead of infection onset results in lagging estimates. Figure 3 demonstrates the effect of increasing population structure on the timing accuracy of ${\overset{ˆ}{ℛ}}_{t}$. Specifically, ${\overset{ˆ}{ℛ}}_{t}<1$ estimated from daily simulated case reports leads true ${ℛ}_{t}<1$ ($\delta_{t}$ is negative) when population structure is strong (Figure 3).

**Figure 4:**
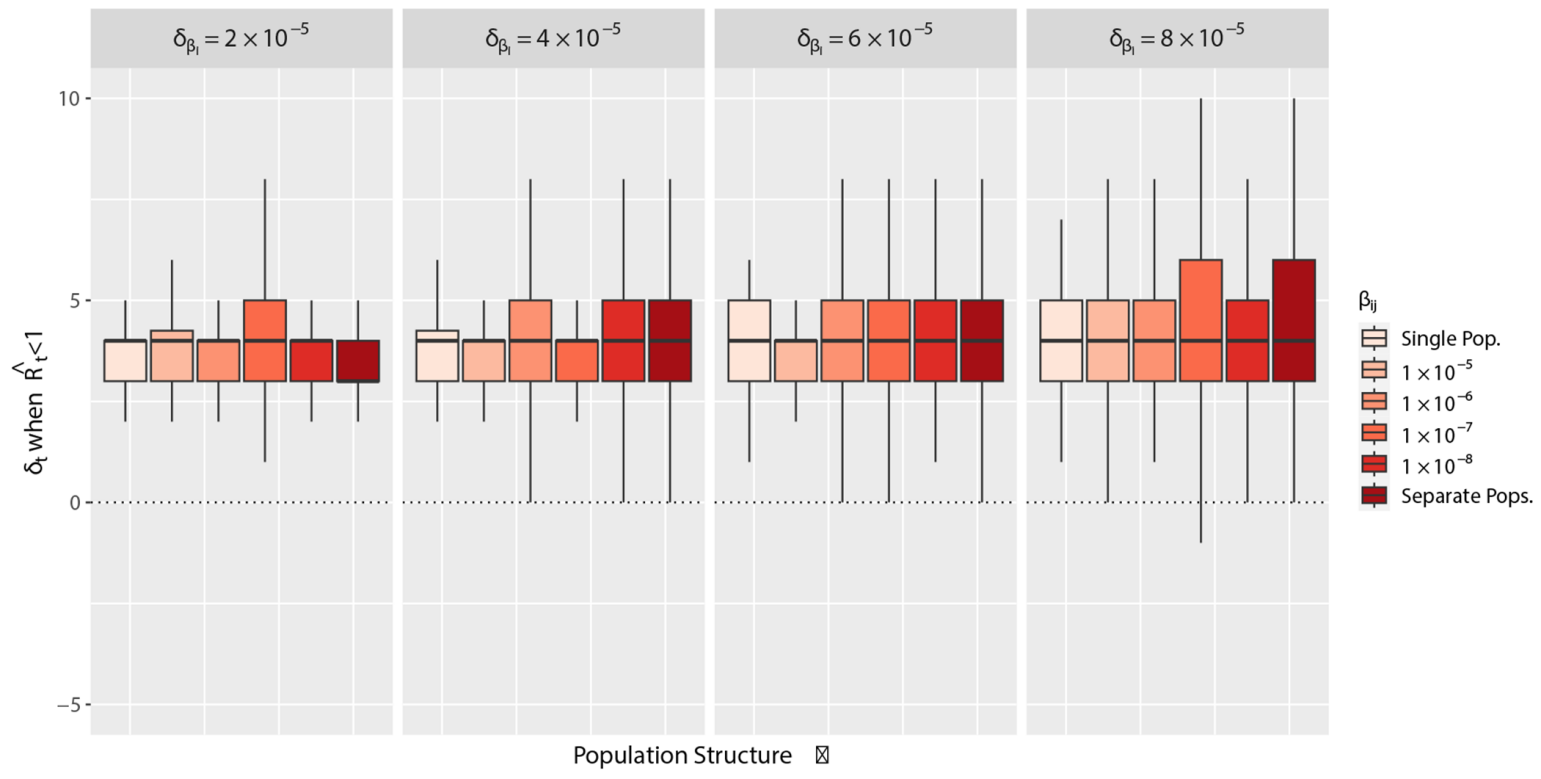
Results from simulated data estimating ${\overset{ˆ}{ℛ}}_{t}$ from daily and weekly aggregated case reports with EpiEstim over varying degrees of population structure. Population structure varies along the x-axis by increasing $\delta_{\beta_{i}}$, the difference $\beta_{j}-\beta_{i}$, and $\beta_{ij}$, the rate of mixing. Within each panel, the lightest colored box represents a single, randomly mixing population where $\delta_{\beta_{i}}=0$ and $\beta_{ij}=\beta_{i}=\beta_{j}$, and the colored boxes correspond to different values of $\beta_{ij}$ or $\beta_{ij}=0$ for separate populations. $\delta_{t}$ on the y-axis is the difference in time points (t) in days of the epidemic time series between ${\overset{ˆ}{ℛ}}_{t}<1$ from weekly minus daily reports.

Next, we determine the timing accuracy of ${\overset{ˆ}{ℛ}}_{t}$ estimated from temporally aggregated data compared to estimates from daily data (Figure 4). Using simulated daily and weekly case report data, Figure 4 shows the difference in time points within the time series measure in days, $\delta_{t}$, when ${\overset{ˆ}{ℛ}}_{t}<1$ as population structure increases. We find that ${\overset{ˆ}{ℛ}}_{t}$ from temporally aggregated data using the methods available in EpiEstim lag behind estimates from daily incidence data with a median $\delta_{t}$ of 4 days. Population structure affects the variance of temporal estimates, but does not further bias the timing when ${\overset{ˆ}{ℛ}}_{t}<1$ beyond the lag time of 4 days (Figure 4).

**Figure 5:**
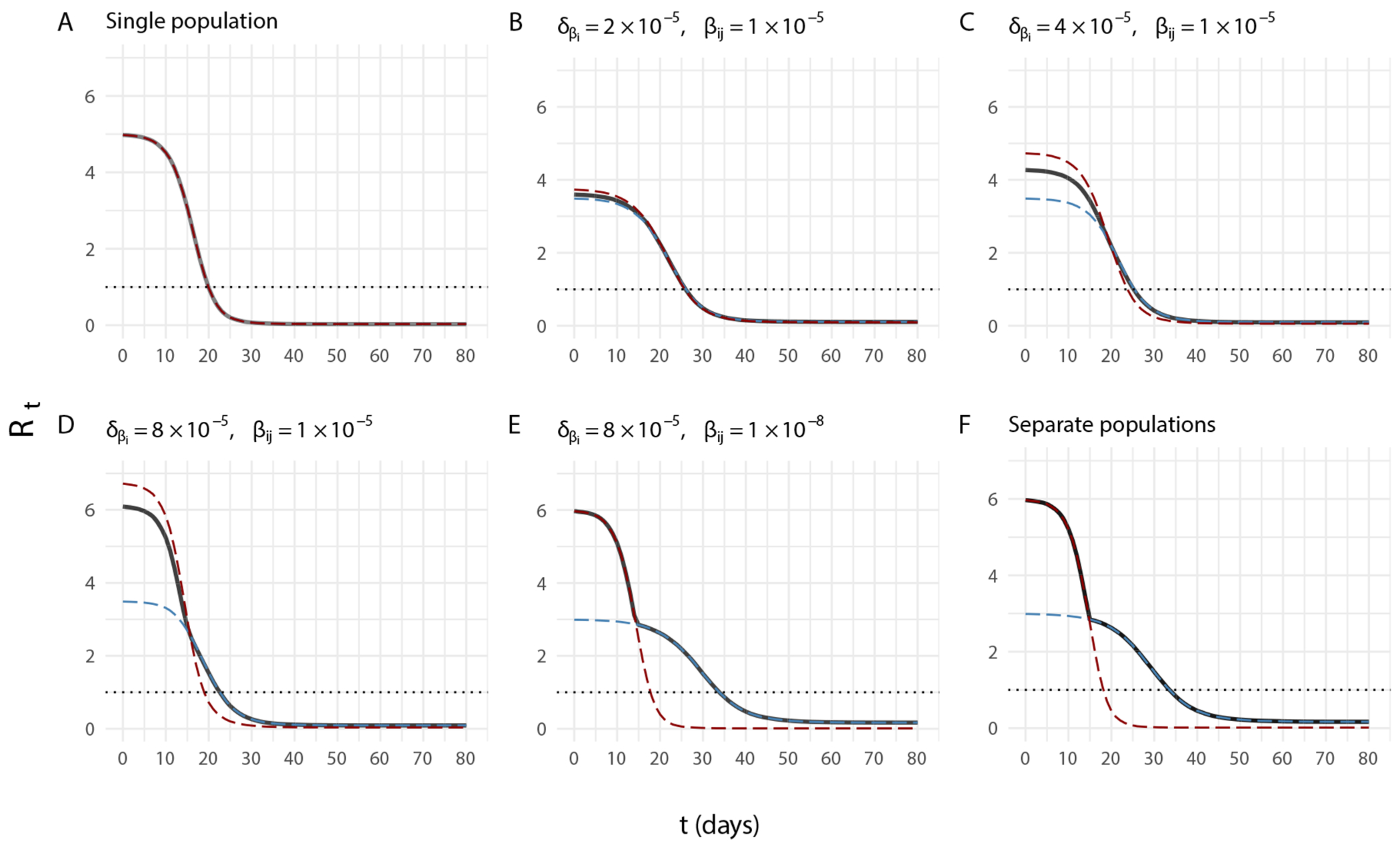
True ${ℛ}_{t}$ for the overall population (black highlighted lines), and true ${ℛ}_{i_{t}}$ (lagging subpopulation; blue dashed lines) and ${ℛ}_{j_{t}}$ (leading subpopulation; red dashed lines) computed from Equations (2) and (4), respectively, and simulated data with parameter values given in Table 1 and specified in each panel. Populations structure increases from (A–F) by changing the values $\delta_{\beta_{i}}$ and $\beta_{ij}$. In the single population $\delta_{\beta_{i}}=0$ and $\beta_{ij}=\beta_{i}=\beta_{j}$. In the separate populations $\delta_{\beta_{i}}=8\times 10^{-5}$ and $\beta_{ij}=0$. Values of $\delta_{\beta_{i}}$ and $\beta_{ij}$ in panels (B–E) are specified.

When population structure is present, the subpopulation transmission dynamics influence ${\overset{ˆ}{ℛ}}_{t}$ and generate the observed bias. Figure 5 shows the relationship between $\left\{{ℛ}_{i_{t}},{ℛ}_{j_{t}}\right\}$ computed from Equation (4) and ${ℛ}_{t}$ computed from Equation (2) over varying degrees of structure. In Figure 5, we can make two observations. First, the decreasing rate of ${ℛ}_{t}$ is abruptly halted due to the decline in suscpetibles in subpopulation j leading to subpopulation i being the dominant outbreak. This effect is evident from the square root term in Equation (2). Second, ${ℛ}_{t}$ approaches the maximal value of $\left\{{ℛ}_{i_{t}},{ℛ}_{j_{t}}\right\}$ as cross-transmission $\left(\beta_{ij}\right)$ approaches zero. This is relevant not only for the temporal accuracy of ${\overset{ˆ}{ℛ}}_{t}$, but also the magnitude of the estimates.

**Figure 6:**
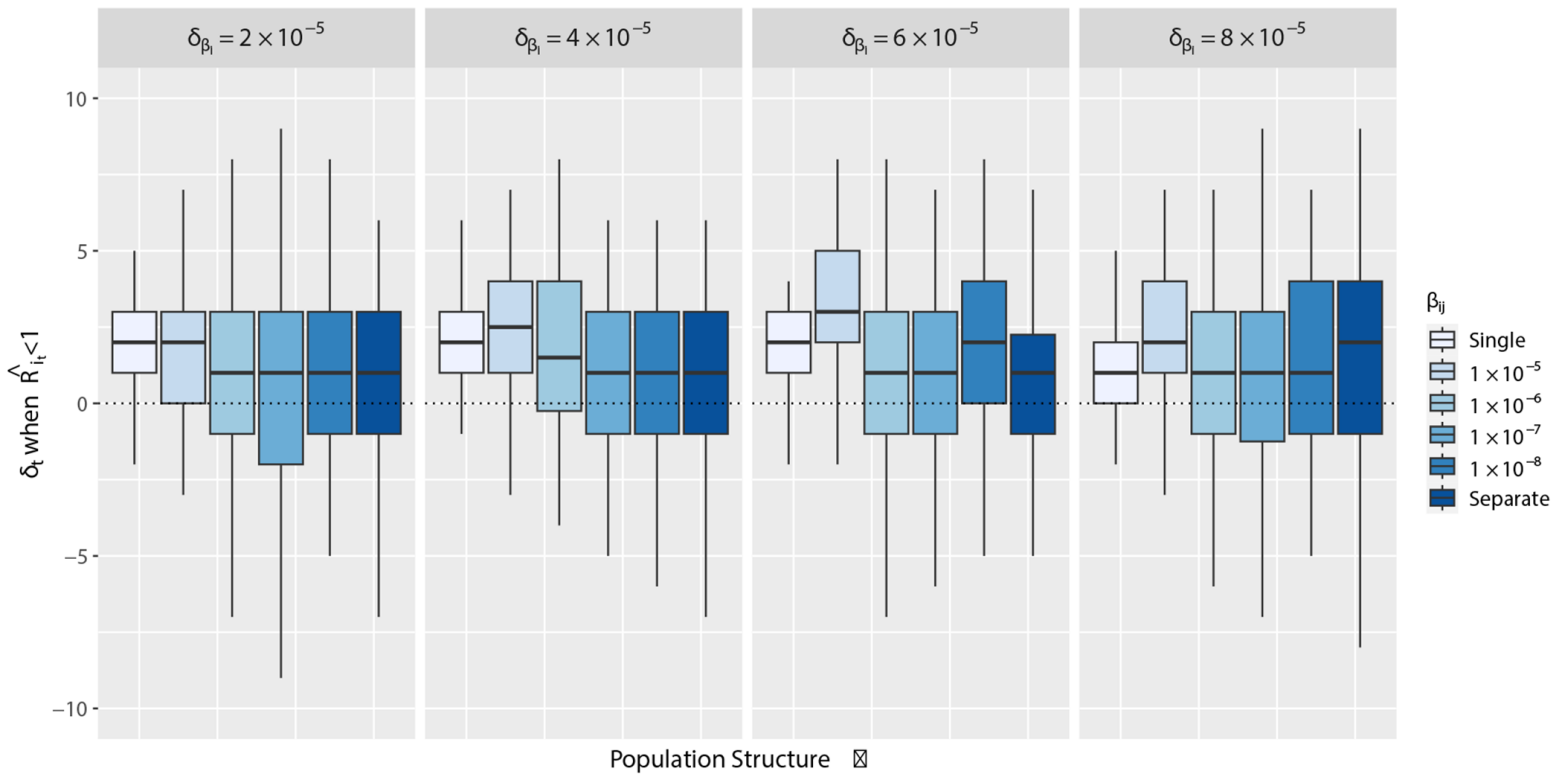
Results from simulated data computing true ${ℛ}_{t}$ and ${\overset{ˆ}{ℛ}}_{i_{t}}$ (from subpopulation i only, the lagging subpopulation) with EpiEstim over varying degrees of population structure. Population structure varies along the x-axis by increasing $\delta_{\beta_{i}}$, the difference $\beta_{j}-\beta_{i}$, and $\beta_{ij}$, the rate of mixing. Within each panel, the lightest colored box represents a single, randomly mixing population where $\delta_{\beta_{i}}=0$ and $\beta_{ij}=\beta_{i}=\beta_{j}$, and the colored boxes correspond to different values of $\beta_{ij}$ or $\beta_{ij}=0$ for separate populations. $\delta_{t}$ on the y-axis is the difference in time points (t) in days of the epidemic time series between ${\overset{ˆ}{ℛ}}_{i_{t}}<1$ minus ${ℛ}_{t}<1$.

These observations lead to a practical solution to recover the timing accuracy when population structure is present. When applying EpiEstim to populations with non-random mixing between subgroups, we demonstrate the subpopulation with the lagging outbreak crosses the ${\overset{ˆ}{ℛ}}_{t}<1$ threshold at approximately the same time point as true ${ℛ}_{t}$. As the subpopulation epidemics diverge (e.g., Figure 5B–F), using the lagging outbreak to estimate ${\overset{ˆ}{ℛ}}_{t}<1$ becomes more important, and the approximation to ${ℛ}_{t}$ improves as $\beta_{ij}$ approaches zero. ${ℛ}_{t}$ has a noticeable inflection where it begins following ${ℛ}_{i_{t}}$. For practical application, Figure 6 shows the results from simulations comparing the timing when true ${ℛ}_{t}<1$ to ${\overset{ˆ}{ℛ}}_{i_{t}}<1$ (subpopulation i has the lagging outbreak) assuming the user of EpiEstim now has data on incidence within each subgroup. If this data is available, our results show that using the subpopulation with the lagging outbreak to determine when ${\overset{ˆ}{ℛ}}_{i_{t}}<1$ removes the bias caused by “hidden” population structure. Using this approximation, the overall bias has a median $\delta_{t}=2$ days, the same value as for a single population.

## Discussion

We have developed a two-population model with a specified generation interval distribution to demonstrate the impact of population structure on ${ℛ}_{t}$ estimation methodology relying on the renewal equation. Our findings rest on the plausible assumption that not all human populations mix randomly and different rates of interactions can have a profound affect on the spread of infectious disease [14]. When using EpiEstim to estimate ${\overset{ˆ}{ℛ}}_{t}$, the resulting time point when ${\overset{ˆ}{ℛ}}_{t}<1$ will be premature if population structure is present but “hidden”. This bias remains unchanged whether incidence is reported daily or aggregated over longer time scales. Thus, if real-time ${\overset{ˆ}{ℛ}}_{t}$ estimates are used to inform decisions, for example when to lift control measures, and these estimates have a temporal bias, it might be possible for an epidemic to re-ignite. Although it is impossible to report disease incidence accounting for all possible heterogeneities within larger populations, in many cases population structure, especially when subpopulations sizes are proportionally large, can be observed alongside incidence [e.g., 21, 31–33].

Our real-world example of COVID-19 incidence reported to Whitman County demonstrates when our findings and recommendations would have practical application. The population in Whitman County has two defined subpopulations, the WSU students and the surrounding community, and this information has been reported alongside incidence. In this case, population structure should be considered when applying the methods of EpiEstim. Our results suggest that the time point when ${\overset{ˆ}{ℛ}}_{t}<1$ from EpiEstim for the lagging outbreak can be used to approximate the time point when ${\overset{ˆ}{ℛ}}_{t}<1$ for the total population, assuming the transmission rates remained approximately constant throughout the epidemic. In Whitman County during the fall 2020 COVID-19 outbreak, the time point when ${\overset{ˆ}{ℛ}}_{t}<1$ for the Whitman community residents would have been the most appropriate estimate to inform public health decisions about when the outbreak ceased in the overall population.

The reproductive number, ℛ, has long been a valuable metric in responding to epidemics [14, 34]. Although new technologies and computational methods (e.g., machine learning) to predict the spread of infectious disease are currently being developed and implemented, classic tools such as EpiEstim and other renewal-equation-based methods are useful to a broader audience. As renewal-equation-based methods continue to be applied in a variety of contexts, the EpiEstim R package [10] included, it is best to continue to inform users of the possible sources of bias as they draw conclusions and make decisions based on these estimates. The results we provide here demonstrate EpiEstim should be prudently applied to outbreak data from structured populations. If data on subgrouping is available along with disease incidence, this information can be used with the existing EpiEstim R software to produce temporally unbiased estimates. Infectious disease continues to be a pervasive threat, thus increasing the functionality of existing statistical tools for public health response will help us prepare for the future.

## Figures and Tables

**Table 1:** Symbols, definitions, parameter values, and their source if applicable used in simulations to vary the effect of population structure.

Symbol	Definition	Value/range	Source
*N*	Total pop. size	35,039	[28], [29]
$\phi_{i}$	Prop. pop. *i*	0.6	[28]
$\phi_{j}$	Prop. pop. *j*	0.4	[29]
$\beta_{i}$	Transmission rate *i*	4 × 10^−5^	NA
$\beta_{j}$	Transmission rate *j*	[6 × 10^−5^, 12 × 10^−5^]	NA
$\beta_{ij}$	Cross-transmission rate	[1 × 10^−5^, 1 × 10^−8^]	NA
σ	Latency rate	1/3.59	[30]
γ	Recovery rate	1/3.56	[30]
ρ	Testing rate	0.1	[30]
k	Dispersion parameter	2	NA
$E_{i0},E_{j0}$	Initial exposed	20	[30]
$I_{i0},I_{j0}$	Initial infected	18	[30]

## Data Availability

All data, Mathematica Notebooks and R code can be found at github.com/erinclancey/EpiEstim-Timing.

## References

[1] Cori A, Ferguson NM, Fraser C, Cauchemez S. A new framework and software to estimate time-varying reproduction numbers during epidemics. Am J Epidemiol 2013;178:1505–12. 24043437 10.1093/aje/kwt133 PMC3816335

[2] Nash RK, Nouvellet P, Cori A. Real-time estimation of the epidemic reproduction number: scoping review of the applications and challenges. PLoS Digit Health 2022;1:e0000052. 36812522 10.1371/journal.pdig.0000052 PMC9931334

[3] Nash RK, Bhatt S, Cori A, Nouvellet P. Estimating the epidemic reproduction number from temporally aggregated incidence data: a statistical modelling approach and software tool. PLoS Comput Biol 2023;19:e1011439. 37639484 10.1371/journal.pcbi.1011439 PMC10491397

[4] Nouvellet P, Cori A, Garske T, Blake IM, Dorigatti I, Hinsley W, A simple approach to measure transmissibility and forecast incidence. Epidemics 2018;22:29–35. 28351674 10.1016/j.epidem.2017.02.012 PMC5871640

[5] Wallinga J, Teunis P. Different epidemic curves for severe acute respiratory syndrome reveal similar impacts of control measures. Am J Epidemiol 2004;160:509–16. 15353409 10.1093/aje/kwh255 PMC7110200

[6] Bettencourt LM, Ribeiro RM. Real time Bayesian estimation of the epidemic potential of emerging infectious diseases. PLoS One 2008;3:e2185. 18478118 10.1371/journal.pone.0002185 PMC2366072

[7] Lytras T. Estimate epidemic effective reproduction number in a Bayesian framework [R package bayEStim version 0.0.1] [Internet]. Available from: https://github.com/thlytras/bayEStim.

[8] Scire J, Huisman JS, Grosu A, Angst DC, Lison A, Li J, estimateR: an R package to estimate and monitor the effective reproductive number. BMC Bioinf 2023;24:310. 10.1186/s12859-023-05428-4 PMC10416499 37568078

[9] Abbott S, Hellewell J, Thompson RN, Sherratt K, Gibbs HP, Bosse NI, Estimating the time-varying reproduction number of SARS-CoV-2 using national and subnational case counts. Wellcome Open Res 2020;5:112.

[10] Cori A, Cauchemez S, Ferguson NM, Fraser C, Dahlqwist E, Demarsh PA, Package ‘EpiEstim’. Vienna Austria: CRAN; 2020, vol 13.

[11] R Core Team. R: a language and environment for statistical computing. Vienna, Austria; 2023. Available from: https://www.R-project.org/.

[12] Gostic KM, McGough L, Baskerville EB, Abbott S, Joshi K, Tedijanto C, Practical considerations for measuring the effective reproductive number, Rt. PLoS Comput Biol 2020;16:e1008409. 33301457 10.1371/journal.pcbi.1008409 PMC7728287

[13] Watts DJ, Muhamad R, Medina DC, Dodds PS. Multiscale, resurgent epidemics in a hierarchical metapopulation model. Proc Natl Acad Sci USA 2005;102:11157–62. 16055564 10.1073/pnas.0501226102 PMC1183543

[14] Lloyd-Smith JO, Schreiber SJ, Kopp PE, Getz WM. Superspreading and the effect of individual variation on disease emergence. Nature 2005;438:355–9. 16292310 10.1038/nature04153 PMC7094981

[15] White LF, Archer B, Pagano M. Determining the dynamics of influenza transmission by age. Emerg Themes Epidemiol 2014;11:1–10. 24656239 10.1186/1742-7622-11-4 PMC3997935

[16] Vazquez A. Epidemic outbreaks on structured populations. J Theor Biol 2007;245:125–9. 17097683 10.1016/j.jtbi.2006.09.018

[17] Fraser C. Estimating individual and household reproduction numbers in an emerging epidemic. PLoS One 2007;2:e758. 17712406 10.1371/journal.pone.0000758 PMC1950082

[18] Klinkenberg D, Fraser C, Heesterbeek H. The effectiveness of contact tracing in emerging epidemics. PLoS One 2006;1:e12. 17183638 10.1371/journal.pone.0000012 PMC1762362

[19] Delamater PL, Street EJ, Leslie TF, Yang YT, Jacobsen KH. Complexity of the basic reproduction number (R0). Emerg Infect Dis 2019;25:1. 10.3201/eid2501.171901 PMC6302597 30560777

[20] Davies NG, Klepac P, Liu Y, Prem K, Jit M, Eggo RM. Age-dependent effects in the transmission and control of COVID-19 epidemics. Nat Med 2020;26:1205–11. 32546824 10.1038/s41591-020-0962-9

[21] Bharti N, Lambert B, Exten C, Faust C, Ferrari M, Robinson A. Large university with high COVID-19 incidence is not associated with excess cases in non-student population. Sci Rep 2022;12:3313. 35228585 10.1038/s41598-022-07155-x PMC8885693

[22] Painter I, Huynh G, Lavista Ferres JM, Etzioni R, Richardson BA, Thakkar N, SitRep 15: COVID-19 transmission across Washington state; 2020. Available from: https://iazpvnewgrp01.blob.core.windows.net/source/archived/WA_Situation_Report_15_COVID-19_transmission_across_Washington_State.pdf.

[23] Diekmann O, Heesterbeek J, Roberts MG. The construction of next-generation matrices for compartmental epidemic models. J R Soc Interface 2010;7:873–85. 19892718 10.1098/rsif.2009.0386 PMC2871801

[24] Britton T, Scalia Tomba G. Estimation in emerging epidemics: biases and remedies. J R Soc Interface 2019;16:20180670. 30958162 10.1098/rsif.2018.0670 PMC6364646

[25] Champredon D, Dushoff J, Earn DJ. Equivalence of the Erlang-distributed SEIR epidemic model and the renewal equation. SIAM J Appl Math 2018;78:3258–78.

[26] Wolfram Research Inc. Mathematica. Champaign, Illinois: Wolfram Research, Inc.; 2021. Available from: https://www.wolfram.com/mathematica.

[27] King AA, Nguyen D, Ionides EL. Statistical inference for partially observed Markov processes via the R package pomp. J Stat Softw 2016;69:1–43.

[28] Washington State Office of Financial Management. State of Washington 2021 population trends; 2021. Available from: https://ofm.wa.gov/sites/default/files/public/dataresearch/pop/april1/ofm_april1__poptrends.pdf.

[29] Office of Strategy, Planning, and Analysis. Total student enrollment – Washington State University; 2022. Available from: https://ir.wsu.edu/total-student-enrollment/.

[30] Pei S, Kandula S, Shaman J. Differential effects of intervention timing on COVID-19 spread in the United States. Sci Adv 2020;6:eabd6370. 33158911 10.1126/sciadv.abd6370 PMC7821895

[31] Riley S, Fraser C, Donnelly CA, Ghani AC, Abu-Raddad LJ, Hedley AJ, Transmission dynamics of the etiological agent of SARS in Hong Kong: impact of public health interventions. Science 2003;300:1961–6. 12766206 10.1126/science.1086478

[32] Thomas R. Estimated population mixing by country and risk cohort for the HIV/AIDS epidemic in Western Europe. J Geogr Syst 2001;3:283–301.

[33] Hué S, Pillay D, Clewley JP, Pybus OG. Genetic analysis reveals the complex structure of HIV-1 transmission within defined risk groups. Proc Natl Acad Sci USA 2005;102:4425–9. 15767575 10.1073/pnas.0407534102 PMC555492

[34] Achaiah NC, Subbarajasetty SB, Shetty RM. R0 and re of COVID-19: can we predict when the pandemic outbreak will be contained? Indian J Crit Care Med Peer-Rev Off Publ Indian Soc Crit Care Med 2020;24:1125. 10.5005/jp-journals-10071-23649 PMC7751056 33384521

